# Structure of a ribonucleotide reductase R2 protein radical

**DOI:** 10.1126/science.adh8160

**Published:** 2023-10-05

**Authors:** Hugo Lebrette, Vivek Srinivas, Juliane John, Oskar Aurelius, Rohit Kumar, Daniel Lundin, Aaron S. Brewster, Asmit Bhowmick, Abhishek Sirohiwal, In-Sik Kim, Sheraz Gul, Cindy Pham, Kyle D. Sutherlin, Philipp Simon, Agata Butryn, Pierre Aller, Allen M. Orville, Franklin D. Fuller, Roberto Alonso-Mori, Alexander Batyuk, Nicholas K. Sauter, Vittal K. Yachandra, Junko Yano, Ville R. I. Kaila, Britt-Marie Sjöberg, Jan Kern, Katarina Roos, Martin Högbom

**Affiliations:** 1 Department of Biochemistry and Biophysics, Stockholm University, Arrhenius Laboratories for Natural Sciences, Stockholm, Sweden; 2 Laboratoire de Microbiologie et Génétique Moléculaires, Centre de Biologie Intégrative, CNRS, Université Toulouse III, Toulouse, France; 3 MAX IV Laboratory, Lund University, Lund, Sweden; 4 Molecular Biophysics and Integrated Bioimaging Division, Lawrence Berkeley National Laboratory, Berkeley, CA, USA; 5 Diamond Light Source Ltd, Harwell Science and Innovation Campus, Didcot, United Kingdom; 6 Research Complex at Harwell, Harwell Science and Innovation Campus, Didcot, United Kingdom; 8 LCLS, SLAC National Accelerator Laboratory, Menlo Park, CA, USA; 9 Department of Cell and Molecular Biology, Uppsala University, Uppsala, Sweden

## Abstract

Aerobic ribonucleotide reductases (RNRs) initiate synthesis of DNA building blocks by generating a free radical within the R2 subunit; the radical is subsequently shuttled to the catalytic R1 subunit through proton-coupled electron transfer (PCET). We present a high-resolution room temperature structure of the class Ie R2 protein radical captured by x-ray free electron laser serial femtosecond crystallography. The structure reveals conformational reorganization to shield the radical and connect it to the translocation path, with structural changes propagating to the surface where the protein interacts with the catalytic R1 subunit. Restructuring of the hydrogen bond network, including a remarkably short O–O interaction of 2.41 Å, likely tunes and gates the radical during PCET. These structural results help explain radical handling and mobilization in RNR and have general implications for radical transfer in proteins.

Uncontrolled or unmitigated free radicals can cause damage to cells; however, radicals are also essential to numerous metabolic pathways and enzyme-mediated chemistry (1, 2). Ribonucleotide reductase (RNR) is an archetypal radical enzyme, and the tyrosyl radical (Y•) in the R2 subunit from *Escherichia coli* was the first stable protein radical to be observed 50 years ago (3). RNR provides the only pathway for *de novo* synthesis of deoxyribonucleotides and represents a drug target for both cancer and infectious diseases (4, 5). Aerobic RNR (class I) depends on the ferritin-like R2 subunit to generate a catalytic radical in an oxygen dependent reaction; the radical must be transferred back-and-forth with the catalytic R1 subunit, which performs the ribonucleotide reduction (6). Radical translocation between the subunits proceeds via reversible long-range proton-coupled electron transfer (PCET) in a transient R1-R2 complex (7). Radical transfer initiation involves redox-induced structural changes in R2 (8, 9), conformational gating (10), short-range proton transfer (11) coupled to long-distance electron transfer (12), and regulation by R1 (7). Recently, the structure of an R1-R2 holocomplex was determined by cryo-EM (7) providing a picture of the long-range radical transfer pathway. However, atomic resolution snapshots of the radical state and the conformational gating taking place at the R2 active site remain unresolved.

Most R2 proteins harbor a conserved tyrosine residue oxidized to Y• by an oxygen-activated metal center in the catalytically active state. In a recently discovered active metal-free R2 subclass, denoted R2e, this tyrosine residue is post-translationally *meta*-hydroxylated to a 3,4-dihydroxyphenylalanine (DOPA) which serves as the radical-harboring residue (13, 14). Oxygen activation of R2e and metal-containing R2 proteins display analogous pathways consisting of a non-activated state, a catalytically active radical state and a radical-lost ground state (Fig. 1A).

From an experimental point of view, R2e from *Mesoplasma florum* (*Mf*R2) represents an attractive model to study active radical states in RNRs. Its radical state is metal-independent which simplifies sample preparation and excludes partial-occupancy or mismetalation of the metal site often encountered with metalloenzymes *in vitro* (15). In addition, in absence of protein R1 its radical state is remarkably stable with no decay observed after more than six hours at 25°C (13). However, as with any radical state, it is expected to be highly sensitive to photoreduction as x-ray radiation damage generates free radicals that spread through protein crystals (16). This obstacle renders the structural characterization of a protein radical state using standard x-ray crystallography methods unfeasible (17, 18). Though many crystal structures of iron- and manganese-containing R2 proteins from different organisms have been obtained, they describe either the non-activated state, the radical-lost ground state (often referred to as the “met” state) or partially reduced states. Crystal structures of R2e have been solved from two different organisms (13, 14), likewise showing signs of X-ray induced photoreduction.

Discrepancies between R2 crystal structures and spectroscopic data of radical states have been observed, leading to contradictory theoretical models regarding the conformation of Y• and its environment (8, 19–21). In the present study, by rapid protein production, microcrystallization and X-ray free-electron laser (XFEL) serial femtosecond crystallography, using the diffraction-before-destruction principle (22), we have determined the atomic structure of *Mf*R2 in the active radical state at room temperature. Compared with the structure of the radical-lost ground state, also presented here, the radical state structure reveals a notably short hydrogen bond and a critical rearrangement of conserved residues upon acquisition of the radical. Based on these two distinct states, we propose a mechanism for structural recognition of the radical state and a model for redox-coupled conformational gating as a prologue to the radical transfer. This mechanism defines central aspects of the PCET process and may be conserved in aerobic RNRs.

## Structure of the radical-lost ground state of *Mf*R2

**Fig. 1:**
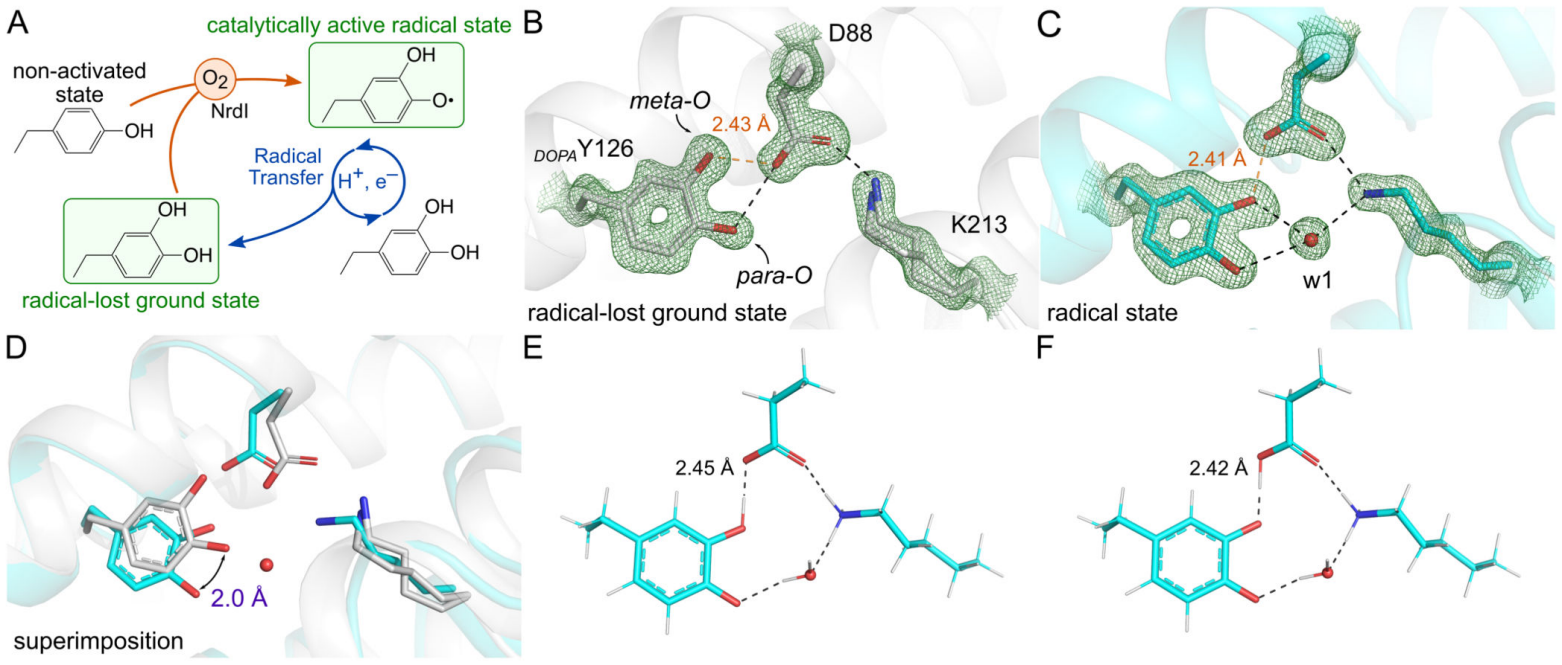
Radical state and radical-lost ground state of *Mf*R2. (**A**) Outline of the proposed activation pathway of metal-free R2e, including the non-activated state, catalytically active radical state, and radical-lost ground state (also known as “met” in canonical R2). The two states determined in this work are indicated in green. The structure of R2e in the non-activated state has been determined previously (13, 14). For clarity, chemical reactions are not strictly balanced. (**B**) Structure of *MfR*2 in the radical-lost ground state obtained after chemical-quenching by hydroxyurea (monomer A is shown). (**C**) Structure of *Mf*R2 in the radical state obtained from XFEL serial femtosecond crystallography showing a reorganization of the site compared to the ground state including a coupled movement of the *_DOPA_*Y126-D88 dyad, the presence of a new water w1 and the inward conformation of K213. The short H-bond is highlighted in orange. (**D**) Superimposition of the ground and radical states. The 2-Å displacement of the DOPA *para*-O is marked in purple. Nitrogen and oxygen atoms are shown in blue and red, respectively. Carbons are shown in grey and cyan for the radical-lost ground state and radical state, respectively. Distance between atoms involved in H-bond interactions are in Å. Simulated annealing composite Omit 2*F_o_*−*F_c_* electron density maps are shown in green and contoured at 2 σ. The structural changes are further illustrated in Movie S1. (**E**, **F**) Using quantum mechanical calculations on the XFEL structure, the short H-bond between _*DOPA*_Y126 and D88 can be reproduced by a DOPA radical state with the radical mainly located on the *para*-O and the proton located on *meta*-O (**E**, neutral DOPA radical state) or D88 (**F**, negatively charged DOPA radical state). For clarity, only a subset of residues included in the calculations is presented on the figure (see Fig. S3 for full details).

In the catalytically active radical state, the radical-harboring *meta*-hydroxylated tyrosyl (from here denoted _*DOPA*_Y126•) in *Mf*R2 exhibits a characteristic absorbance peak at 383 nm and colors the protein blue (13). The catalytic radical can be chemically quenched by hydroxyurea, a known RNR radical scavenger used for decades as an antitumor drug (23) proposed to inactivate R2 through PCET (24). Importantly, hydroxyurea causes a reversible inactivation of *Mf*R2 as the enzyme can recover activity upon re-oxidation by NrdI (13). Thus, the protein is not permanently inactivated or damaged but resides in a radical-lost ground state (Fig. 1A).

To ensure an accurate depiction of the radical-lost ground state of *Mf*R2, we solved the crystal structure at 1.35 Å resolution of the protein chemically quenched by incubation with hydroxyurea before crystallization. This treatment abolished the 383-nm absorbance peak and rendered the protein colorless (13). The electron density map clearly shows the post-translational modification in the *meta* position of _*DOPA*_Y126 (Fig. 1B). The two oxygen-containing functional groups of _*DOPA*_Y126 in the *para* and *meta* positions (denoted *para*-O and *meta*-O, respectively) form hydrogen bonds (H-bond) with D88. Strikingly, the *meta*-O is involved in a remarkably short interaction (Fig. 1B). Using END/RAPID error analysis (25), the O–O distance between _*DOPA*_Y126 and D88 was calculated to 2.43 ± 0.04 Å, a length which could correspond to a low-barrier or single-well H-bond (26, 27). In addition, _*DOPA*_Y126 does not interact with any water, nor with K213 whose ε-ammonium group is facing away from _*DOPA*_Y126 (Fig. 1B). Compared to previous R2e structures, differences and alternate conformations are observed throughout the structures, particularly variations in the conformation of _*DOPA*_Y126, D88 and K213 are observed (Fig. S1A).

## Structure of the radical state of *Mf*R2 by serial femtosecond crystallography

To circumvent photoreduction artefacts, we used XFEL serial femtosecond crystallography to determine the structure of the catalytically active radical state of *Mf*R2 at atomic resolution. The active radical-harboring protein was crystallized in batch to produce a suspension of blue microcrystals used to collect room temperature serial femtosecond diffraction data at an XFEL source. The resulting dataset produced a model of the protein at 1.5 Å resolution, and the electron density map allows unambiguous interpretation (Fig. 1C). A short H-bond between _*DOPA*_Y126 *meta*-O and D88 is present with a O–O distance calculated to 2.41 ± 0.05 Å using END/RAPID error analysis (25) (see Methods for details). A short H-bond is observed in both states of the protein and may play a special structural role, as suggested in other cases (28), contributing to maintaining the integrity of the enzyme active site (29). The short H-bond may stabilize the interaction between _*DOPA*_Y126 and D88 in order to ensure that no hydrogen is available to mediate a putative proton transfer from D88 to the _*DOPA*_Y126 *para*-O, which would annul productive PCET. We note that this H-bonding structure results in a situation analogous to canonical R2 proteins where a deprotonated aspartate is involved in metal coordination, and not in proton transfer, thus forcing the latter to occur with a different nearby proton donor, suggested to be a metal-bound water molecule (11). Furthermore, it is tempting to speculate that this short H-bond is involved in redox tuning. By preventing the radical delocalization between the *meta*-O and *para*-O, the DOPA radical becomes electronically more similar to a tyrosyl radical, rather than a DOPA-semiquinone radical, in agreement with previous characterization of *Mf*R2 by electron paramagnetic resonance (EPR) (13).

A striking conformational shift takes place upon radical acquisition between _*DOPA*_Y126 and D88: the dyad undergoes a coupled coplanar (but not coaxial) rotation of ~22°, with an additional rotation of the aromatic ring of ~12° along the C_β_-C_1_ axis. It results in a 2-Å displacement of the *para*-O carrying the main radical spin density, away from D88, and a reorganization of the interaction pattern around _*DOPA*_Y126 (Fig. 1D). The aspartate residue corresponding to D88 in *Mf*R2 is strictly conserved as the N-terminal metal-coordinating residue in Y•-harboring canonical R2 proteins and exhibits redox-induced conformational changes in metal-containing R2 proteins (8, 30, 17, 31). Furthermore, coupled movements of the conserved radical-harboring Y have been observed to be redox-induced in R2b from *Bacillus anthracis* (17). For R2a from *E. coli* (*Ec*R2a), the conserved aspartate is proposed to form a H-bond with the reduced Y in the ground state. In contrast, single-crystal EPR experiments suggest that the Y• rotates away in the radical state, leading to a ~1-Å displacement of the radical-harboring oxygen and breaking the connection with the aspartate (8). A displacement of the Y• could also be hypothesized from discrepancies observed between crystal structures and spectroscopic data for R2 proteins from *Bacillus anthracis* (32), *Salmonella typhimurium* (33), *Corynebacterium ammoniagenes* (30) and mouse (19). This type of rearrangement upon acquisition of the radical is principally similar to what we observe in *Mf*R2 structures, and is less pronounced than movements proposed in other studies, which involve either translation of the main chain or larger Y• displacement by several Å (20, 21, 24).

In addition, a clearly defined water molecule (w1) mediates a new H-bond between _*DOPA*_Y126 and the ε-ammonium group of K213, which displays a different orientation facing towards _*DOPA*_Y126 (Fig. 1C). K213 adopts a single well-defined conformation different from previously published structures of active R2e determined using synchrotron radiation (13) (Fig. S1B). The presence of a water molecule at a position similar to w1 has been observed previously in other RNR systems (34, 35), and seems to be dependent on the redox state (17) (Fig. S2). Moreover, superimposition with structures of R2 proteins shows that the new location of the K213 ε-ammonium group corresponds to the position of another water molecule that is metal-coordinated in canonical R2 proteins (Fig. S2). This water is proposed to transfer a proton to Y• in the conformational gate initiating the PCET (11, 36, 37). Residue K213 was recently suggested by density functional theory to be a proton donor for radical transfer in R2e (38). Therefore, it may represent the water-equivalent proton-donor in the case of R2e, and thus its conformational change could effectuate a comparable conformational gate.

Comparing the structures of the defined radical and radical-lost states, determined here, to previously solved structures of R2e proteins shows that no previous structure fully represents either the radical state or the radical-lost state (Fig. S1). Although the proteins in prior work may have originally crystallized in the ‘active form’, they appear to have suffered different degrees of X-ray induced photoreduction during synchrotron data collection.

## The XFEL structure can be reproduced *in silico* by a radical state

In order to evaluate if the crystal structure obtained by XFEL femtosecond crystallography theoretically corresponds to a radical state, calculations were performed on the crystal structure active site. Based on quantum chemical geometry optimizations, the short interaction between _*DOPA*_Y126 and D88 could be reproduced with the main radical character on the *para*-O. The proton could reside on either _*DOPA*_Y126 or D88, both states produced a short O–O distance (Fig. 1, E and F, fig. S3, A and B). The calculated energy difference of 3 kcal/mol between the two states suggests that both states are accessible, with a slight favor of the proton residing closer to _*DOPA*_Y126•. In addition, various alternative _*DOPA*_Y126 states were modeled by quantum mechanical calculations, none of them agreeing well with the experimental observations. In particular a longer hydrogen bond with D88 is observed when _*DOPA*_Y126 is modeled as a neutral DOPA, a DOPA quinone or with the radical located on the *meta-*O (Fig. S3, C to E). Furthermore, the calculated spin population of _*DOPA*_Y126• in the protein active site models revealed an asymmetric distribution closer to a *meta*-substituted Y• than the fully delocalized character of an *ortho*-semiquinone, based on comparisons with calculated distributions in smaller models. This is fully consistent with spectroscopic data of the radical (13, 14) and indicates a possible role for the short *_DOPA_*Y126-D88 hydrogen bond to destabilize the otherwise potentially too stable semiquinone radical in the protein.

Molecular dynamics (MD) simulation starting from the XFEL structure of the radical state but with induced loss of the radical *in silico*, showed _*DOPA*_Y126 movement with the dynamics dominated by the position of the radical-lost ground state, forming two H-bonds to D88 (Fig. S3, F to H), consistent with the crystal structure. Altogether, our calculations support that the structure obtained by XFEL corresponds to the catalytically active radical state of the *Mf*R2 protein, and are in agreement with previous EPR and UV–vis spectroscopic results (13) showing that the spin density distributes similar as in a *meta*-substituted tyrosyl radical rather than as in an *ortho*-semiquinone.

## Specific protein rearrangements upon radical acquisition

**Fig. 2:**
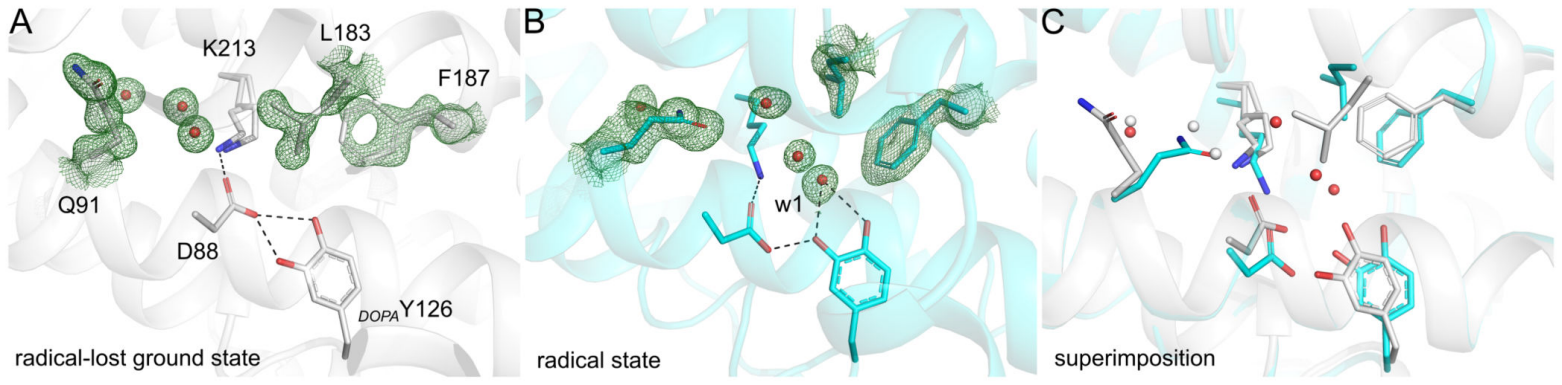
Major conformational changes between the radical-lost ground state and the radical state. (**A**) Structure of *Mf*R2 in the radical-lost ground state obtained after chemical-quenching by hydroxyurea. (**B**) Structure of *Mf*R2 in the radical state obtained from XFEL serial femtosecond data showing reorganization of the site compared to the radical-lost ground state, including conformational changes of Q91, L183 and F187. In the radical state, Q91 displaces two water molecules, breaking the water H-bond network toward _*DOPA*_Y126. Structural movements of L183 and F187 leave space for 3 water molecules interacting with D88, _*DOPA*_Y126 and K213. Simulated annealing composite Omit 2*F_o_*−*F_c_* electron density maps are shown in green and contoured at 1.5 σ. (**C**) Superimposition of the structures of *Mf*R2 in the radical state (cyan) and radical-lost ground state (grey).

The radical acquisition in *Mf*R2 leads to two major protein rearrangements that are of particular interest as they can directly be implicated in radical generation, stabilization and transfer. The first major protein rearrangement takes place within the activating-oxidant path. The channel connecting the NrdI flavin cofactor to the R2b metal site (39, 40), proposed as the O_2_^•–^ route for activation, seems to be conserved in R2e (14). In the radical-lost ground state of *Mf*R2, in place of the metal center present in canonical R2, a continuous chain of well-defined H-bonded water molecules creates the link between the putative oxidant route and the _*DOPA*_Y126-D88 dyad. In contrast, in the radical state of *Mf*R2, this water network is disrupted by Q91 which undergoes a large sidechain flip towards D88 (Fig. 2, Movie S1). In the radical-lost ground state, Q91 is involved in a H-bond network conserved in the R2b subclass (Q70 in R2b from *E. coli*), which lines the channel for oxidant transport to the Mn^II^_2_ active site (40). Our data suggest that Q91 could play a key role as it obstructs the putative oxidant channel in the radical state, preventing radical quenching by further oxidant access to the active site.

The second major protein rearrangement upon radical acquisition concerns the PCET route. In the immediate vicinity of the side chain of _*DOPA*_Y126, residues L183 and F187 undergo large conformational changes in the radical state of *Mf*R2, leaving space for the positioning of three water molecules (including w1) and creating a water-mediated H-bond network between *_DOPA_*Y126, D88, K213 and Q91 (Fig. 2, Movie S1). Residues L183 and F187 belong to helix αE which forms a distorted π-helix conserved across many ferritin superfamily members. The π-helix conformation of helix αE is believed to play a functional role (41, 42), and is known to undergo redox-induced structural rearrangements (17, 30, 34). Residues L183 and F187 are conserved in all R2 proteins, the position of L183 being either F or L (in 91% and 9% of sequences, respectively) and F187 being conserved in 99% of the sequences. In none of the R2 crystal structures solved to date, these residues exhibit conformations similar to those in the *Mf*R2 radical state (Fig. S4). In the first solved structure of R2, it was noted that the radical-harboring tyrosyl oxygen was surrounded by a conserved hydrophobic pocket formed by residues F208, F212 and I234 in *Ec*R2a (equivalents to L183, F187 and I209 in *Mf*R2, respectively) (43). The major function of these residues was proposed through mutational studies to contribute to the tyrosyl radical stability by insulating the radical-harboring tyrosyl oxygen (44). Our observation of hitherto unseen movements of these radical shielding residues further implicates them in radical control and suggests their involvement in gating of the ribonucleotide reductase PCET mechanism. The specific local rearrangements at the radical site also translate to movements of the protein backbone and global structural changes of the protein scaffold protruding to the R1 interaction surface and the radical transfer path (Movie S2). It has previously been shown that the active R1-R2 complex exhibits tighter binding after radical initiation (45–47). We propose that these global structural changes observed in R2 provide a mechanism by which the R1-R2 binding properties can be modulated during the catalytic cycle.

## Model of conformational gating for radical transfer initiation

**Fig. 3:**
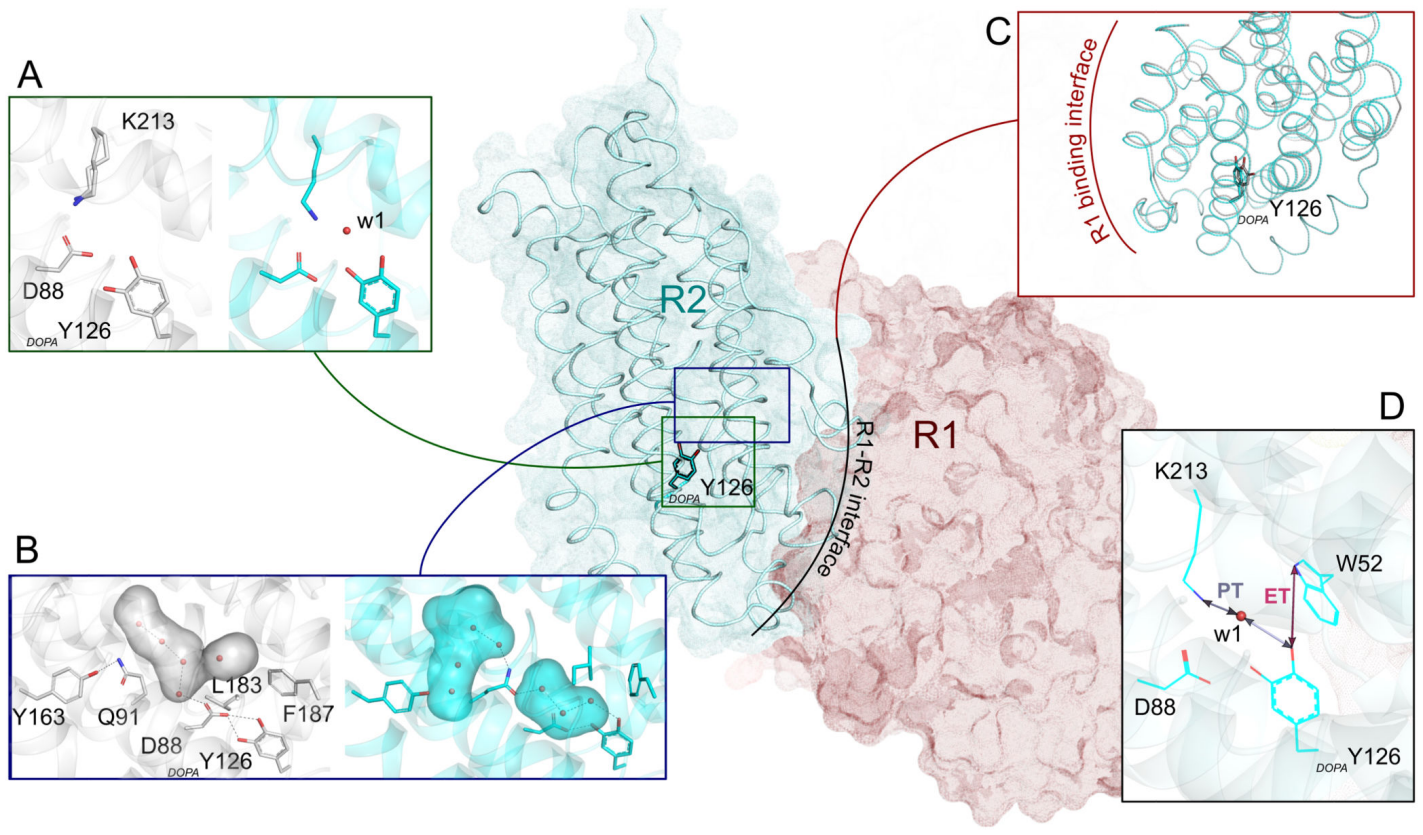
Model of conformational gating and radical transfer during PCET in R2e. (**A**) Oxidation of the radical-lost ground state _*DOPA*_Y126 (grey) displaces _*DOPA*_Y126-D88 and introduces a water molecule (w1) to facilitate the proton transfer from the redirected amino group of K213 (cyan). (**B**) Concurrently, the cavity surrounding the generated _*DOPA*_Y126 radical is reshaped, shown in surface representation (radical-lost state and radical state in grey and cyan, respectively). Primarily, Q91 flips away from Y163 to block access of any further oxidant from NrdI through the R2-NrdI channel and, L183 and F187 flip to reshape the cavity and facilitate electron transfer from W52 to _*DOPA*_Y126. (**C**) Structure superposition of *Mf*R2 in the radical (cyan) and radical-lost (grey) states at the R1 binding interface. Reshaping of the pockets surrounding the _*DOPA*_Y126 leads to conformational changes protruding to the R2-R1 interaction surface and binding to protein R1 completes the PCET pathway. (**D**) PCET is initiated by a proton transfer (PT) from the amino group of K213 to _*DOPA*_Y126 mediated by w1, and simultaneously an electron transfer from W52 to _*DOPA*_Y126, thus translocating the radical towards the active site C394 of R1. R1 (represented in red) is modelled in complex with the radical-state *Mf*R2 using the cryo-EM structure of the R1-R2 holocomplex from *E. coli* (PDB ID: 6w4x) (7).

In *Mf*R2, the catalytically active radical state and the radical-lost ground state are interconvertible by quenching the radical and through NrdI-mediated re-oxidation of _*DOPA*_Y126 (Fig. 1A). Based on our structural data, we propose a model of the conformational gating orchestrated by R2 after radical acquisition, which is a prelude to the radical translocation to the R1 subunit (Fig. 3). This model proceeds in three steps. Firstly, the oxidation of _*DOPA*_Y126 leads to a repulsion between its *para*-O and D88 due to the removal of the H-bonding hydrogen atom, resulting in the 2-Å displacement of the _*DOPA*_Y126 *para*-O (Fig. 3A). Secondly, this triggers a cascade of structural changes to shield the radical and prepare its transfer: Q91 blocks the access to further oxidant, L183 and F187 reshape the insulating pocket around the radical, and water w1 connects the _*DOPA*_Y126 radical-carrying oxygen with the PCET route (Fig. 3B). This also leads to global structural rearrangements of protein R2, including the R2-R1 interaction surface and binding of protein R1 (Fig. 3C, Movie S2). Thirdly, the formation of the R1-R2 complex results in the ordering of the full R2 C-terminal tail at the R1-R2 interface (as demonstrated in (7)), completing the electron transfer path and inducing the injection of an electron to reduce _*DOPA*_Y126•, e.g. via the conserved W52 and/or Y325 (corresponding to W48 and Y356 in *Ec*R2a), as previously suggested (12) (Fig. 3D). Coupled to this event, a proton transfer from K213 to _*DOPA*_Y126• occurs via the water w1 (as proposed in (38)), and initiates the long-range radical translocation. Figure 3 summarizes the proposed steps of conformational gating in R2e. The interaction with R1 is modeled based on a superposition of the cryo-EM structure of the R1-R2 holocomplex from *E. coli* (7).

This conformational gating model for initiating the radical translocation could be common to class I RNR systems. In PCET, proton transfer occurs through H-bond networks and requires the proton donor and acceptor to be within a standard ~2.8 Å H-bond distance (48). As a displacement of the radical-harboring Y upon radical acquisition is also observed by spectroscopy in other R2 proteins (8, 19, 30, 32, 33), an intermediary may be required between the metal-bound water and Y•. In *Mf*R2 the additional water w1 gained upon radical acquisition represents the missing piece of the puzzle, connecting the radical to K213 (Fig. 3D). We note that an analogous binding site for water has been observed in other R2 proteins (Fig. S2), and that the ε-ammonium group of K213 is located at the position of the metal-bound water in metal-containing R2s (Fig. S2). Therefore, we speculate that, similarly as in R2e, a water in this position links the radical to the metal-bound water proposed to be the proton donor in canonical R2 proteins and gates the first proton transfer to initiate radical translocation to R1.

## Supplementary Material

Movie S1

Movie S2

Supplementary Materials

## Data Availability

Atomic coordinates and structure factors have been deposited in the Protein Data Bank (PDB) with the following codes: catalytically active radical state solved by XFEL, 8bt3; radical-lost ground state, 8bt4. *In silico* models and output are available at the SciLifeLab Data Repository (49).

## One sentence summary

XFEL serial femtosecond crystallography was used to obtain an atomic resolution snapshot of a ribonucleotide reductase R2 protein radical.

## References

[1] Högbom M, Sjöberg B-M, Berggren G (2020). eLS.

[2] Stubbe J, Nocera DG (2021). Radicals in Biology: Your Life Is in Their Hands. J Am Chem Soc.

[3] Ehrenberg A, Reichard P (1972). Electron spin resonance of the iron-containing protein B2 from ribonucleotide reductase. J Biol Chem.

[4] Miret-Casals L, Baelo A, Julián E, Astola J, Lobo-Ruiz A, Albericio F, Torrents E (2018). Hydroxylamine Derivatives as a New Paradigm in the Search of Antibacterial Agents. ACS Omega.

[5] Xie Y, Wang Y, Xu Z, Lu Y, Song D, Gao L, Yu D, Li B, Chen G, Zhang H, Feng Q (2022). Preclinical validation and phase I trial of 4-hydroxysalicylanilide, targeting ribonucleotide reductase mediated dNTP synthesis in multiple myeloma. J Biomed Sci.

[6] Banerjee R, Srinivas V, Lebrette H, Harris JR, Marles-Wright J (2022). Macromolecular Protein Complexes IV.

[7] Kang G, Taguchi AT, Stubbe J, Drennan CL (2020). Structure of a trapped radical transfer pathway within a ribonucleotide reductase holocomplex. Science.

[8] Högbom M, Galander M, Andersson M, Kolberg M, Hofbauer W, Lassmann G, Nordlund P, Lendzian F (2003). Displacement of the tyrosyl radical cofactor in ribonucleotide reductase obtained by single-crystal high-field EPR and 1.4-Å x-ray data. Proc Natl Acad Sci.

[9] Offenbacher AR, Vassiliev IR, Seyedsayamdost MR, Stubbe J, Barry BA (2009). Redox-linked structural changes in ribonucleotide reductase. J Am Chem Soc.

[10] Yokoyama K, Uhlin U, Stubbe J (2010). A hot oxidant, 3-NO2Y122 radical, unmasks conformational gating in ribonucleotide reductase. J Am Chem Soc.

[11] Wörsdörfer B, Conner DA, Yokoyama K, Livada J, Seyedsayamdost M, Jiang W, Silakov A, Stubbe J, Bollinger JM, Krebs C (2013). Function of the diiron cluster of Escherichia coli class Ia ribonucleotide reductase in proton-coupled electron transfer. J Am Chem Soc.

[12] Reece SY, Seyedsayamdost MR (2017). Long-range proton-coupled electron transfer in the Escherichia coli class Ia ribonucleotide reductase. Essays Biochem.

[13] Srinivas V, Lebrette H, Lundin D, Kutin Y, Sahlin M, Lerche M, Eirich J, Branca RMM, Cox N, Sjöberg B-M, Högbom M (2018). Metal-free ribonucleotide reduction powered by a DOPA radical in Mycoplasma pathogens. Nature.

[14] Blaesi EJ, Palowitch GM, Hu K, Kim AJ, Rose HR, Alapati R, Lougee MG, Kim HJ, Taguchi AT, Tan KO, Laremore TN (2018). Metal-free class Ie ribonucleotide reductase from pathogens initiates catalysis with a tyrosine-derived dihydroxyphenylalanine radical. Proc Natl Acad Sci U S A.

[15] Cotruvo JA, Stubbe J (2012). Metallation and mismetallation of iron and manganese proteins in vitro and in vivo: the class I ribonucleotide reductases as a case study. Metallomics.

[16] Garman EF (2010). Radiation damage in macromolecular crystallography: what is it and why should we care?. Acta Crystallogr D Biol Crystallogr.

[17] Grāve K, Lambert W, Berggren G, Griese JJ, Bennett MD, Logan DT, Högbom M (2019). Redox-induced structural changes in the di-iron and di-manganese forms of Bacillus anthracis ribonucleotide reductase subunit NrdF suggest a mechanism for gating of radical access. J Biol Inorg Chem.

[18] Sigfridsson KGV, Chernev P, Leidel N, Popović-Bijelić A, Gräslund A, Haumann M (2013). Rapid X-ray photoreduction of dimetal-oxygen cofactors in ribonucleotide reductase. J Biol Chem.

[19] Strand KR, Karlsen S, Kolberg M, Røhr AK, Görbitz CH, Andersson KK (2004). Crystal structural studies of changes in the native dinuclear iron center of ribonucleotide reductase protein R2 from mouse. J Biol Chem.

[20] Barry BA, Chen J, Keough J, Jenson D, Offenbacher A, Pagba C (2012). Proton Coupled Electron Transfer and Redox Active Tyrosines: Structure and Function of the Tyrosyl Radicals in Ribonucleotide Reductase and Photosystem II. J Phys Chem Lett.

[21] Offenbacher AR, Burns LA, Sherrill CD, Barry BA (2013). Redox-linked conformational control of proton-coupled electron transfer: Y122 in the ribonucleotide reductase β2 subunit. J Phys Chem B.

[22] Chapman HN, Fromme P, Barty A, White TA, Kirian RA, Aquila A, Hunter MS, Schulz J, DePonte DP, Weierstall U, Doak RB (2011). Femtosecond X-ray protein nanocrystallography. Nature.

[23] Saban N, Bujak M (2009). Hydroxyurea and hydroxamic acid derivatives as antitumor drugs. Cancer Chemother Pharmacol.

[24] Offenbacher AR, Barry BA (2020). A Proton Wire Mediates Proton Coupled Electron Transfer from Hydroxyurea and Other Hydroxamic Acids to Tyrosyl Radical in Class Ia Ribonucleotide Reductase. J Phys Chem B.

[25] Lang PT, Holton JM, Fraser JS, Alber T (2014). Protein structural ensembles are revealed by redefining X-ray electron density noise. Proc Natl Acad Sci U S A.

[26] Perrin CL, Nielson JB (1997). “Strong” hydrogen bonds in chemistry and biology. Annu Rev Phys Chem.

[27] Perrin CL (2010). Are short, low-barrier hydrogen bonds unusually strong?. Acc Chem Res.

[28] Nichols DA, Hargis JC, Sanishvili R, Jaishankar P, Defrees K, Smith EW, Wang KK, Prati F, Renslo AR, Woodcock HL, Chen Y (2015). Ligand-Induced Proton Transfer and Low-Barrier Hydrogen Bond Revealed by X-ray Crystallography. J Am Chem Soc.

[29] Kemp MT, Lewandowski EM, Chen Y (2021). Low barrier hydrogen bonds in protein structure and function. Biochim Biophys Acta Proteins Proteomics.

[30] Cox N, Ogata H, Stolle P, Reijerse E, Auling G, Lubitz W (2010). A tyrosyl-dimanganese coupled spin system is the native metalloradical cofactor of the R2F subunit of the ribonucleotide reductase of Corynebacterium ammoniagenes. J Am Chem Soc.

[31] Logan DT, Su XD, Aberg A, Regnström K, Hajdu J, Eklund H, Nordlund P (1996). Crystal structure of reduced protein R2 of ribonucleotide reductase: the structural basis for oxygen activation at a dinuclear iron site. Struct Lond Engl 1993.

[32] Torrents E, Sahlin M, Biglino D, Gräslund A, Sjöberg B-M (2005). Efficient growth inhibition of Bacillus anthracis by knocking out the ribonucleotide reductase tyrosyl radical. Proc Natl Acad Sci U S A.

[33] Galander M, Uppsten M, Uhlin U, Lendzian F (2006). Orientation of the tyrosyl radical in Salmonella typhimurium class Ib ribonucleotide reductase determined by high field EPR of R2F single crystals. J Biol Chem.

[34] Eriksson M, Jordan A, Eklund H (1998). Structure of Salmonella typhimurium nrdF ribonucleotide reductase in its oxidized and reduced forms. Biochemistry.

[35] Högbom M, Huque Y, Sjöberg B-M, Nordlund P (2002). Crystal structure of the di-iron/radical protein of ribonucleotide reductase from Corynebacterium ammoniagenes. Biochemistry.

[36] Siegbahn PEM, Eriksson L, Himo F, Pavlov M (1998). Hydrogen Atom Transfer in Ribonucleotide Reductase (RNR). J Phys Chem B.

[37] Stubbe J, Nocera DG, Yee CS, Chang MCY (2003). Radical initiation in the class I ribonucleotide reductase: long-range proton-coupled electron transfer?. Chem Rev.

[38] Zou J, Chen Y, Feng W (2022). Mechanism of DOPA radical generation and transfer in metal-free class Ie ribonucleotide reductase based on density functional theory. Comput Struct Biotechnol J.

[39] John J, Aurelius O, Srinivas V, Saura P, Kim I-S, Bhowmick A, Simon PS, Dasgupta M, Pham C, Gul S, Sutherlin KD (2022). Redox-controlled reorganization and flavin strain within the ribonucleotide reductase R2b-NrdI complex monitored by serial femtosecond crystallography. eLife.

[40] Boal AK, Cotruvo JA, Stubbe J, Rosenzweig AC (2010). Structural basis for activation of class Ib ribonucleotide reductase. Science.

[41] Lundin D, Poole AM, Sjöberg B-M, Högbom M (2012). Use of structural phylogenetic networks for classification of the ferritin-like superfamily. J Biol Chem.

[42] Cooley RB, Arp DJ, Karplus PA (2010). Evolutionary origin of a secondary structure: π-helices as cryptic but widespread insertional variations of α-helices that enhance protein functionality. J Mol Biol.

[43] Nordlund P, Sjöberg BM, Eklund H (1990). Three-dimensional structure of the free radical protein of ribonucleotide reductase. Nature.

[44] Ormö M, Regnström K, Wang Z, Que L, Sahlin M, Sjöberg BM (1995). Residues important for radical stability in ribonucleotide reductase from Escherichia coli. J Biol Chem.

[45] Greene BL, Nocera DG, Stubbe J (2018). Basis of dATP inhibition of RNRs. J Biol Chem.

[46] Minnihan EC, Ando N, Brignole EJ, Olshansky L, Chittuluru J, Asturias FJ, Drennan CL, Nocera DG, Stubbe J (2013). Generation of a stable, aminotyrosyl radical-induced α2β2 complex of Escherichia coli class la ribonucleotide reductase. Proc Natl Acad Sci U S A.

[47] Greene BL, Kang G, Cui C, Bennati M, Nocera DG, Drennan CL, Stubbe J (2020). Ribonucleotide Reductases: Structure, Chemistry, and Metabolism Suggest New Therapeutic Targets. Annu Rev Biochem.

[48] Migliore A, Polizzi NF, Therien MJ, Beratan DN (2014). Biochemistry and theory of proton-coupled electron transfer. Chem Rev.

[49] Lebrette H, Roos K, Kaila V, Högbom M (2023). QM/MM and MD result files for the MfR2e system. SciLifeLab Data Repository.

[50] Kabsch W (2010). XDS. Acta Crystallogr D Biol Crystallogr.

[51] Afonine PV, Grosse-Kunstleve RW, Echols N, Headd JJ, Moriarty NW, Mustyakimov M, Terwilliger TC, Urzhumtsev A, Zwart PH, Adams PD (2012). Towards automated crystallographic structure refinement with phenix.refine. Acta Crystallogr D Biol Crystallogr.

[52] Sierra RG, Batyuk A, Sun Z, Aquila A, Hunter MS, Lane TJ, Liang M, Yoon CH, Alonso-Mori R, Armenta R, Castagna JC (2019). The Macromolecular Femtosecond Crystallography Instrument at the Linac Coherent Light Source. J Synchrotron Radiat.

[53] Fuller FD, Gul S, Chatterjee R, Burgie ES, Young ID, Lebrette H, Srinivas V, Brewster AS, Michels-Clark T, Clinger JA, Andi B (2017). Drop-on-demand sample delivery for studying biocatalysts in action at X-ray free-electron lasers. Nat Methods.

[54] Brewster AS, Waterman DG, Parkhurst JM, Gildea RJ, Young ID, O’Riordan LJ, Yano J, Winter G, Evans G, Sauter NK (2018). Improving signal strength in serial crystallography with DIALS geometry refinement. Acta Crystallogr Sect Struct Biol.

[55] Zeldin OB, Brewster AS, Hattne J, Uervirojnangkoorn M, Lyubimov AY, Zhou Q, Zhao M, Weis WI, Sauter NK, Brunger AT (2015). Data Exploration T oolkit for serial diffraction experiments. Acta Crystallogr D Biol Crystallogr.

[56] Sauter NK (2015). XFEL diffraction: developing processing methods to optimize data quality. Synchrotron J Radiat.

[57] Hattne J, Echols N, Tran R, Kern J, Gildea RJ, Brewster AS, Alonso-Mori R, Glö C, Hellmich J, Laksmono H, Sierra RG (2014). Accurate macromolecular structures using minimal measurements from X-ray free-electron lasers. Nat Methods.

[58] McCoy AJ, Grosse-Kunstleve RW, Adams PD, Winn MD, Storoni LC, Read RJ (2007). Phaser crystallographic software. J Appl Crystallogr.

[59] Emsley P, Lohkamp B, Scott WG, Cowtan K (2010). Features and development of Coot. Acta Crystallogr D Biol Crystallogr.

[60] Liebschner D, Afonine PV, Baker ML, Bunkóczi G, Chen VB, Croll TI, Hintze B, Hung LW, Jain S, McCoy AJ, Moriarty NW (2019). Macromolecular structure determination using X-rays, neutrons and electrons: recent developments in Phenix. Acta Crystallogr Sect Struct Biol.

[61] Williams CJ, Headd JJ, Moriarty NW, Prisant MG, Videau LL, Deis LN, Verma V, Keedy DA, BJ Hintze, Chen VB, Jain S (2018). MolProbity: More and better reference data for improved all-atom structure validation. Protein Sci Publ Protein Soc.

[62] Ho BK, Gruswitz F (2008). HOLLOW: generating accurate representations of channel and interior surfaces in molecular structures. BMC Struct Biol.

[63] Gurusaran M, Shankar M, Nagarajan R, Helliwell JR, Sekar K (2014). Do we see what we should see? Describing non-covalent interactions in protein structures including precision. IUCrJ.

[64] Kumar KSD, Gurusaran M, Satheesh SN, Radha P, Pavithra S, Thulaa Tharshan KPS, Helliwell JR, Sekar K (2015). Online_DPI: a web server to calculate the diffraction precision index for a protein structure. J Appl Crystallogr.

[65] Huang J, Rauscher S, Nawrocki G, Ran T, Feig M, de Groot BL, Grubmüller H, MacKerell AD (2017). CHARMM36m: an improved force field for folded and intrinsically disordered proteins. Nat Methods.

[66] Phillips JC, Hardy DJ, Maia JDC, Stone JE, Ribeiro JV, Bernardi RC, Buch R, Fiorin G, Hénin J, Jiang W, McGreevy R (2020). Scalable molecular dynamics on CPU and GPU architectures with NAMD. J Chem Phys.

