# Supplementary material for: Structure of a ribonucleotide reductase R2 protein radical
Source: Science. Author manuscript; Available in PMC 2024 Jan 9. (PMC7615503; doi:10.1126/science.adh8160)
Supplement: Supplementary Materials [file EMS192781-supplement-Supplementary_Materials.pdf]

Science
AAAS

Supplementary Materials for

Structure of a ribonucleotide reductase R2 protein radical

Hugo Lebrette[1,2]†, Vivek Srinivas[1]†, Juliane John[1], Oskar Aurelius[1,3], Rohit Kumar[1], Daniel Lundin[1], Aaron S. Brewster[4], Asmit Bhowmick[4], Abhishek Sirohiwal[1], In-Sik Kim[4], Sheraz Gul[4], Cindy Pham[4], Kyle D. Sutherlin[4], Philipp Simon[4], Agata Butryn[5,6,7], Pierre Aller[5,6], Allen M. Orville[5,6], Franklin D. Fuller[8], Roberto Alonso-Mori[8], Alexander Batyuk[8], Nicholas K. Sauter[4], Vittal K. Yachandra[4], Junko Yano[4], Ville R. I. Kaila[1], Britt-Marie Sjöberg[1], Jan Kern[4]*, Katarina Roos[9,10] & Martin Högbom[1]*

*Corresponding authors: hugo.lebrette@univ-tlse3.fr, jfkern@lbl.gov, hogbom@dbb.su.se

The PDF file includes:

Materials and Methods
Figures S1 to S5
Tables S1
References (50–66)

Other Supplementary Materials for this manuscript include the following:

Movies S1 to S6

Materials and Methods

No statistical methods were used to predetermine sample size. The experiments were not randomized, and the investigators were not blinded to allocation during experiments and outcome assessment.

Protein production and purification

Mesoplasma florum R2 was produced and purified using the plasmid pET28Mf*nrdFI* as previously described (*13*). In brief, *M. florum nrdF* and *nrdI* genes were PCR-amplified from genomic DNA of *M. florum* L1 (NCTC 11704, obtained from Public Health England). All codons translating to tryptophan in the *nrdF* gene were mutated from TGA to TGG to correct for the difference in codon usage between *M. florum* and *Escherichia coli*. Genes *nrdF* and *nrdI* were cloned together into a pET-28a plasmid (Novagen) previously modified, with the thrombin cleavage site replaced by a tobacco etch virus (TEV) protease cleavage site. The generated plasmid pET28Mf*nrdFI* features the *nrdF* gene directly downstream of the N-terminal TEV-cleavable 6xHis-tag, followed by a ribosome-binding site ahead of the *nrdI* gene. *E. coli* BL21(DE3) (NEB) carrying the plasmid pRAREcamR (Novagen) was transformed with the pET28Mf*nrdFI* plasmid. Cultures were grown in LB medium (Formedium) at 37 °C, supplemented with kanamycin and chloramphenicol, in a benchtop bioreactor system (Harbinger). When an optical density at 600 nm of 0.7 was reached, expression was induced with 0.5 mM isopropyl β-D-1-thiogalactopyranoside and allowed to continue overnight at room temperature. Then, cells were harvested by centrifugation and stored at −80 °C. The His-tagged *Mf*R2 protein was purified at 4°C *via* immobilized metal ion affinity chromatography (IMAC) and size-exclusion chromatography (SEC). A cell pellet was resuspended in buffer A (25 mM HEPES-Na pH 7.0, 20 mM imidazole and 300 mM NaCl) and disrupted by high-pressure homogenization (EmulsiFlex-C3). The lysate was cleared by centrifugation, applied to a nickel-nitrilotriacetic acid agarose (Protino) gravity flow column, and washed extensively with buffer B (buffer A containing 40 mM imidazole). Protein was eluted using buffer C (buffer A containing 250 mM imidazole), concentrated using Vivaspin centrifugal concentrators (molecular weight cut-off of 30 kDa, Sartorius), and applied to a HiLoad 16/60 Superdex 200 prep grade SEC column (GE Healthcare) equilibrated in a final buffer of 25 mM HEPES-Na pH 7.0 and 50 mM NaCl. The fractions corresponding to *Mf*R2 were pooled and subjected to TEV protease cleavage. A reverse IMAC step was performed to isolate the His-tag free *Mf*R2, which was subsequently concentrated. Protein purity and radical content were verified by SDS-PAGE and UV-visible spectrophotometry (**Fig. S5**). Finally, the protein was aliquoted, flash-frozen and stored at −80 °C.

*Mf*R2 radical-quenching by hydroxyurea treatment

Prior to crystallization, 50 µl of 0.6 mM blue, radical-harboring, active *Mf*R2 was incubated for 24 h at room temperature with 100 mM of either hydroxyurea or urea. Hydroxyurea is a known RNR radical scavenger. Urea was used as a control, to confirm that the radical quenching step was not the result of unintended protein denaturation by hydroxyurea. Afterwards, hydroxyurea and urea were washed out by buffer exchange steps on a Vivaspin Turbo 4 concentrator, molecular weight cut-off 30 kDa (Sartorius), and *Mf*R2 was concentrated to 25 mg ml^{-1} for crystallization. The protein retained its blue color after incubation with urea but became colorless when incubated with hydroxyurea, confirming that radical quenching occurred only upon hydroxyurea treatment.

Crystallization of *Mf*R2 in the radical-lost state for synchrotron data collection

*Mf*R2 was crystallized at a concentration of 25 mg ml^{-1} using the sitting-drop vapor diffusion method in MRC 2-well crystallization plates (Swissci) using a mosquito nanolitre pipetting robot (TTP Labtech) and a reservoir volume of 50 µl. The reservoir condition was 175 mM calcium acetate, 100 mM ammonium sulphate and 20% (w/v) polyethylene glycol (PEG) 3350. The protein to reservoir volume ratio was 0.2:0.2 µl. Cuboidal blue-colored crystals grow within 2 days at 21 °C. To accelerate the crystallization process, crystal microseeds were added to the crystallization drop. A crystal microseed stock was prepared by resuspending a crystal of *Mf*R2 into a 1.5-ml tube containing 50 µl of reservoir solution and a 3.2-mm PTFE bead (Saint-Gobain) kept on ice, and crushing it by successive vortexing cycles of 30 s. For *Mf*R2 treated with hydroxyurea, cuboidal colorless crystals appear within 24 h at 21°C (dimensions of approximately 100*100*100 μm^3). Before data collection at a synchrotron source, crystals were cryoprotected with a solution made of reservoir solution supplemented with 20% glycerol and flash-frozen in liquid nitrogen.

Synchrotron data collection, data reduction, structure determination

Synchrotron X-ray diffraction data were collected at 100 K at the beamline X06SA (PXI) of the Swiss Light Source (Paul Scherrer Institute, Villigen, Switzerland). Complementary X-ray diffraction data were collected at the beamlines PROXIMA 1 and PROXIMA 2A of the SOLEIL synchrotron (Saint-Aubin, France). Data reduction and scaling were carried out using XDS (*50*). The high-resolution cut-off of the dataset corresponding to the radical-lost state of *Mf*R2 was determined based on a combination of I/σ(I), R_{meas} and $CC_{1/2}$. The crystal belongs to the space group *C*2 with unit cells similar to *Mf*R2 structures that we previously solved (*13*). Phases were retrieved by running rigid body refinement in phenix.refine (*51*) using a curated model with reset B-factors based on the atomic coordinates of the DOPA form of *Mf*R2 (PDB ID: 6gp2) (*13*) defining each of the two chains present in the asymmetric unit as separate groups.

Crystallization for XFEL data collection

In order to provide a fresh crystal microseed stock, *Mf*R2 was crystallized using the sitting-drop vapor diffusion method in MRC Maxi crystallization plates (Swissci), by mixing a volume of 2 µl of a protein solution at 25 mg ml^{-1} with 2 µl of reservoir solution consisting of 200 mM calcium acetate, 10 mM ammonium sulphate and 20% (w/v) PEG 3350. The crystal microseed stock solution was prepared as described above. The microcrystals used for the XFEL data collection were grown using the batch method in a tube containing 12 µl of *Mf*R2 solution at 13 mg ml^{-1}, 12 µl of crystallization buffer and 0.8 µl of microseed stock solution for rapid crystal formation. The crystallization buffer consisted of 50 mM calcium acetate, 20 mM ammonium sulphate and 20% (w/v) PEG 3350. Tubes were incubated upright at 21°C for 24 h allowing platelike crystals to grow (dimensions of approximately 10*10*5 μm^3), before being pooled and resuspended in crystallization buffer supplemented with 10% (v/v) glycerol and loaded into a Hamilton syringe kept under rotation at 21°C.

XFEL sample delivery and data collection

XFEL data were collected at the Macromolecular Femtosecond Crystallography (MFX) instrument of the Linac Coherent Light Source (LCLS) facility (SLAC National Accelerator Laboratory, USA) (*52*) using X-ray pulses of 35 fs length at an energy of 9.5 keV with 30 Hz

repetition rate and with an average pulse power of 2.5 mJ and a beam size of 4 μm (FWHM), obtained by compound refractive lenses. Sample was delivered at room temperature in small droplets of crystal suspension generated by acoustic droplet ejection from a small reservoir and then transported into the X-ray interaction region by a polyimide belt inside a He enclosure, using the "Drop on Tape" setup described previously (*53*). In short, the crystal suspension continuously flowed from a 1 ml gas tight Hamilton syringe into a 6 μl sample reservoir located underneath a focused acoustic transducer. The sample suspension was delivered at a flow rate of ~9 μl/min. Droplets of 5 nl volume were generated using a 10 MHz transducer (for details see (*53*)) and ejected at 30 Hz in synchrony with the detector readout and XFEL pulse delivery. The polyimide belt was running at a speed of 300 mm/s resulting in an exposure of the formed droplets to the He atmosphere of the enclosure for 0.8 s before being probed by the X-ray pulse. Diffraction data was detected at a rate of 30 Hz on a Rayonix MX340-HS detector in 4 x 4 binning mode at a distance of 144 mm from the interaction point. Data was collected for 35.2 minutes yielding 63350 X-ray shots and out of these 14431 were indexed (23% indexing rate).

XFEL data reduction and structure determination

XFEL data was processed using *cctbx.xfel* and DIALS as described previously (*54*). Prior to processing, we applied joint refinement of the Rayonix detector model against 799 crystal models (in this case from a different sample collected concurrently with R2e), generating a new detector position. Even with this refined position, for these data we initially had difficulty determining the space group and unit cell. Briefly, we indexed the data using an initial target unit cell of a = 176.2 Å, b = 53.6 Å, c = 79.2 Å, α = γ = 90°, β = 108.6° and space group *C*2, since this was the presumed isoform of the crystals. Unexpectedly, the crystal unit cell appeared to be different, as suggested by the low indexing rate of the crystal hits. Re-processing the data in P1 with no target cell improved the indexing rate fivefold. After using the program *cluster.unit_cell* (*55*) to identify likely cells based on clustering in P1, we recognized that the unit cell had similarity to an unpublished synchrotron dataset in *C*2, unit cell a = 143 Å, b = 46 Å, c = 58 Å, α = γ = 90°, β = 113°. Using this as an indexing target improved indexing rate another 15% and from these results we were able to solve the structure. We merged 14431 images to 1.50 Å resolution using *cxi.merge*, part of the *cctbx.xfel* package (*56*). To reduce the effects of merging unmeasured signal, we computed a resolution cut-off for each image using I/σ(I) estimates to determine the resolution limits of each crystal (errors were estimated using the Ha14 model described in (*54*, *57*)). We also applied post-refinement for each image, in which the crystal orientation, Wilson B factor, and scale factor are refined, the difference between the scaled, partiality-corrected reflection intensities and the model intensities. The partiality correction inflates the reflection intensities to their full equivalents, analogous to the intensities that would have been measured if the crystal had been rotated through the Ewald sphere. The crystal structure was solved using Phaser (*58*) by molecular replacement using as search model the chain A of the atomic coordinates of the DOPA form of *Mf*R2 (PDB ID: 6gp2) (*13*). A well-contrasted solution was obtained with one molecule per asymmetric unit in space group *C*2.

Model refinement of the synchrotron and XFEL structures

All crystallographic models were examined and modified using the program *Coot* (*59*) and refined using *phenix.refine* (*51*) in the PHENIX suite (*60*). B-factor refinement was performed using individual anisotropic atomic displacement parameters (ADPs) for protein and ligand atoms, and isotropic ADPs for water molecules. Structure validation was performed with MolProbity (*61*). Simulated annealing composite Omit $2F_{obs}–F_{calc}$ electron density maps were

generated for *Mf*R2e structures in both the radical and the radical-lost ground states using *phenix.composite_omit_map*. The method "anneal" was chosen with 5% of the model omitted in each step and R_{free} reflections removed. **Table S1** was generated with *phenix.table_one* and lists the crystallographic statistics in which the test set represents 5% of the reflections. The Ramachandran statistics are: favored 99.01%, and 99.34% for *Mf*R2 crystal structures of the radical state and radical-lost ground state, respectively. Ramachandran outliers were 0.0% in both structures. Figures 1, 2, 3, S1, S2 and S4, and Movies S1 and S2 were prepared using the PyMOL Molecular Graphics System, version 2 Schrödinger, LLC. Representations of interior surfaces in Fig. 3 were generated using HOLLOW (*62*). Residue conservation was obtained through the alignments of 8924 unique R2 sequences from the database NCBI RefSeq, December 2018 (one sequence per species and subclass). We note that protein produced using the protocol described has a radical content of around 52% (*13*). In the radical state structure, however, we observe a single well-defined conformation. It thus appears that the radical conformational state was preferentially selected during crystallization.

END/RAPID error analysis

The END/RAPID method (*25*) was used to estimate the precision of the distance assignment between the oxygen atoms of $_{DOPA}$Y126 and D88. The central idea behind the END/RAPID method is that we want to describe what would be the consequence of repeating the same measurement of structural data several times in terms of the resulting structural model and from that infer the trustworthiness of different aspects of the final model derived. In order to simulate the repetition of the diffraction experiment we introduce random errors to the intensity measurements (based on error estimates for the intensities) and conduct a separate structural refinement for each of these sets of perturbed intensities, generating a different refined structural model. When this procedure is repeated multiple times, the differences between the various structural models adequately capture the modelling error caused directly by errors in the measured intensities. The END/RAPID method thus makes it feasible to propagate the intensity measurement errors to the errors in the structural model for macromolecules.

In this work, we first perturbed the scaled structure factors (F_{obs} scaled to F_{calc}) by a random amount in the range of $\pm |F_{obs} - F_{calc}|$ (assuming that the maximum error of the measurements is similar to the deviation between the observed and calculated structure factors) for 100 separate trials. This gives us 100 independent datasets to refine against. Since intensity error modelling for XFEL experiments is still an active research area, we chose to use the $\pm |F_{obs} - F_{calc}|$ range as it reflects an upper bound for the possible measurement errors. Next, for each of the 100 separate trials, we also perturbed the starting structural model by a random amount using the *sites.shake* tool in Phenix. Prior to refining against the perturbed data, the restraints for the DOPA molecules were also relaxed (0.2 Å for bond distances and 20 degrees for angles). Subsequently, we carried out independent refinement jobs to obtain 100 refined structural models. The refinement settings for these perturbed datasets were kept to be the same as the original refinement, except for the looser restraints of the DOPA molecule, and the same refinement software was used. From the ensemble of refined models, we calculated the standard deviation of the distance between $_{DOPA}$Y126 *meta*-O (OE2) and OD2 of D88. This standard deviation has been reported in the main text. Scripts for performing the END/RAPID error analysis are available at https://bl831.als.lbl.gov/END/RAPID/end.rapid/Documentation/end.rapid.Manual.htm. We note that because the magnitude of the perturbation of the structure factors are larger than the true experimental errors, we expect the obtained error estimate for the distances to be an upper bound to the true error.

To complement the END/RAPID approach, bond-length errors (σ_l) between the $_{DOPA}$Y126 *meta-O* (OE2) and OD2 of D88 were calculated using equation (4) of reference (*63*): $\sigma_l = (\sigma^2_a + \sigma^2_b)^{1/2}$. Atomic coordinate errors (σ_a and σ_b) were computed using the diffraction precision index server *Online_DPI* (http://cluster.physics.iisc.ernet.in/dpi/) (*64*). For the radical state and radical-lost state structures, bond-length errors are 0.09 Å and 0.06 Å, respectively. Both values are comparable (although higher) with values of standard deviations obtained using END/RAPID (0.05 Å and 0.04 Å, respectively).

Computational details

Quantum chemical calculations were performed using DFT with the B3LYP-D3 functional and Schrodinger's Jaguar suite (Schrödinger Release 2018-3: Jaguar, Schrödinger, LLC, New York, NY, 2018). A model of the active site was constructed including residues as depicted in **Fig. S3**. Geometries were optimized in vacuum with the 6-31g** basis set and keeping alpha-carbons and cap-protons along the backbone fixed, single point energies were calculated in vacuum with the cc-pvtz(-f) basis set, and solvation effects from a pbf calculation in a protein medium with a dielectric constant of 4 and the 6-31g** basis set were added.

Molecular dynamics (MD) simulations were performed to probe the conformational dynamics of the radical-lost state of $_{DOPA}$Y126. To this end, a starting model obtained from XFEL data (radical state) of the dimeric *Mf*R2 was embedded in a (TIP3P) water box with 150 mM NaCl, and simulated in the radical-lost state using the CHARMM36 force field (*65*). The system, comprising 83,435 atoms, was simulated in triplicates in an *NPT* ensemble, with p = 1 atm and T = 310 K using an integration timestep of 1 fs, and treating long-range electrostatics interaction with the Particle Mesh Ewald approach. The MD simulations were carried out using NAMD v. 2.14/3.0 (*66*).

Legends for the movies

Movie S1. Structure of the radical site and structural changes upon radical acquisition. Radical-lost state in white, radical state in blue/magenta. Electron densities for the radical-lost state (blue) and radical state (magenta) (2Fo-Fc maps contoured at 1 σ in both cases). Dots represent van der Waals radii. Movement represents a morph between the two states (implemented in PyMOL using default parameters).

Movie S2. Global structural changes upon radical acquisition and radical transfer path. Radical-lost state in white, radical state in blue/magenta. Movement represents a morph between the two states (implemented in PyMOL using default parameters). Surface of protein R1 is depicted in yellow. R1 binding and the radical transfer path is modeled based on the R1-R2 complex structure of *E. coli* class I RNR (*7*).

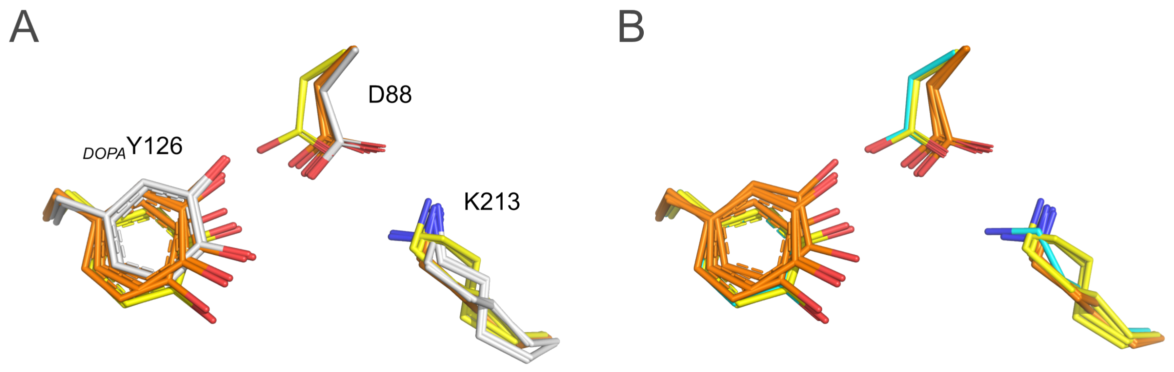

Fig. S1. Superposition of *Mf*R2 structures in the radical and radical-lost states with previously published synchrotron structures of R2e from *Aerococcus urinae* (*Au*R2, PDB ID: 6ebq) (*14*) and *Mesoplasma florum* (*Mf*R2, PDB ID: 6gp2) (*13*). This comparison highlights that all previously published R2e structures, originally crystallized in the 'active form', differ significantly from both the radical and radical-lost states solved in this study, most likely due to X-ray induced photoreduction during synchrotron data collection and/or partial initial occupancy of the radical state. For clarity the figures only include three key residues (*Mf*R2 numbering) and no water molecules but further differences and alternate conformations can be observed throughout the structures. (**A**) *Mf*R2 in the radical-lost state (grey, chains A and B) compared to the previously published synchrotron structures of *Au*R2 (orange, chains A to D) and of *Mf*R2 (yellow, chains A and B). (**B**) *Mf*R2 in the radical state (cyan, chain A) compared to the previously published synchrotron structures of *Au*R2 (orange, chains A to D) and of *Mf*R2 (yellow, chains A and B).

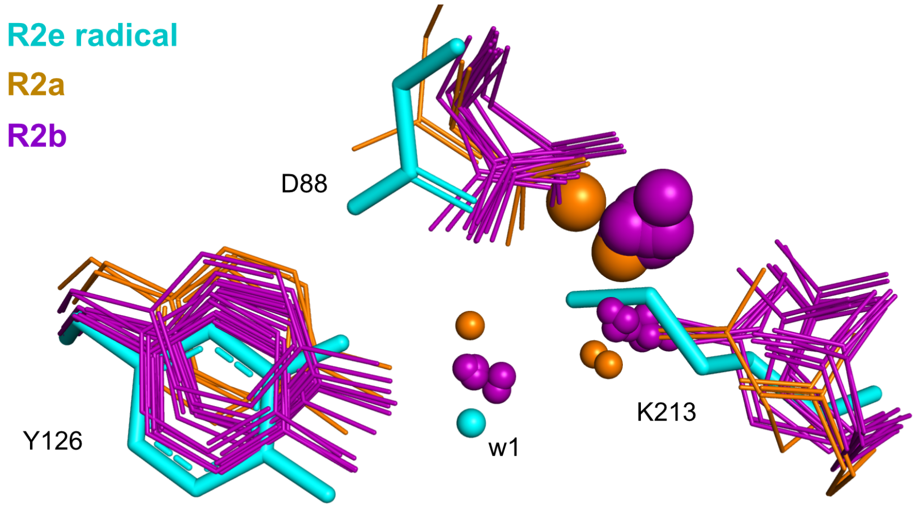

Fig. S2. Superposition of the radical state of *Mf*R2 with representative R2a and R2b structures. Structures were aligned using PyMOL and selected for containing a di-metal center and either having a water in a 2 Å radius around w1 or the ε-ammonium group of K213 or both (which is only the case for 1kgn). R2a structures are shown in orange, R2b in purple and the *Mf*R2e radical structure is shown in cyan. D88, Y126 and K213 or the *Mf*R2e radical structure are shown in sticks and the homolog residues in all other structures as lines. Metals are shown as big spheres, waters as small spheres. PDB IDs of the selected structures are: R2a: 1w68, 1mxr, 7bet; R2b: 3mjo, 1kgp, 1kgn, 1uzr, 3n37, 2r2f, 6qob, 6tqy, 6qo7, 4bmo, 4bmu, 4bmt.

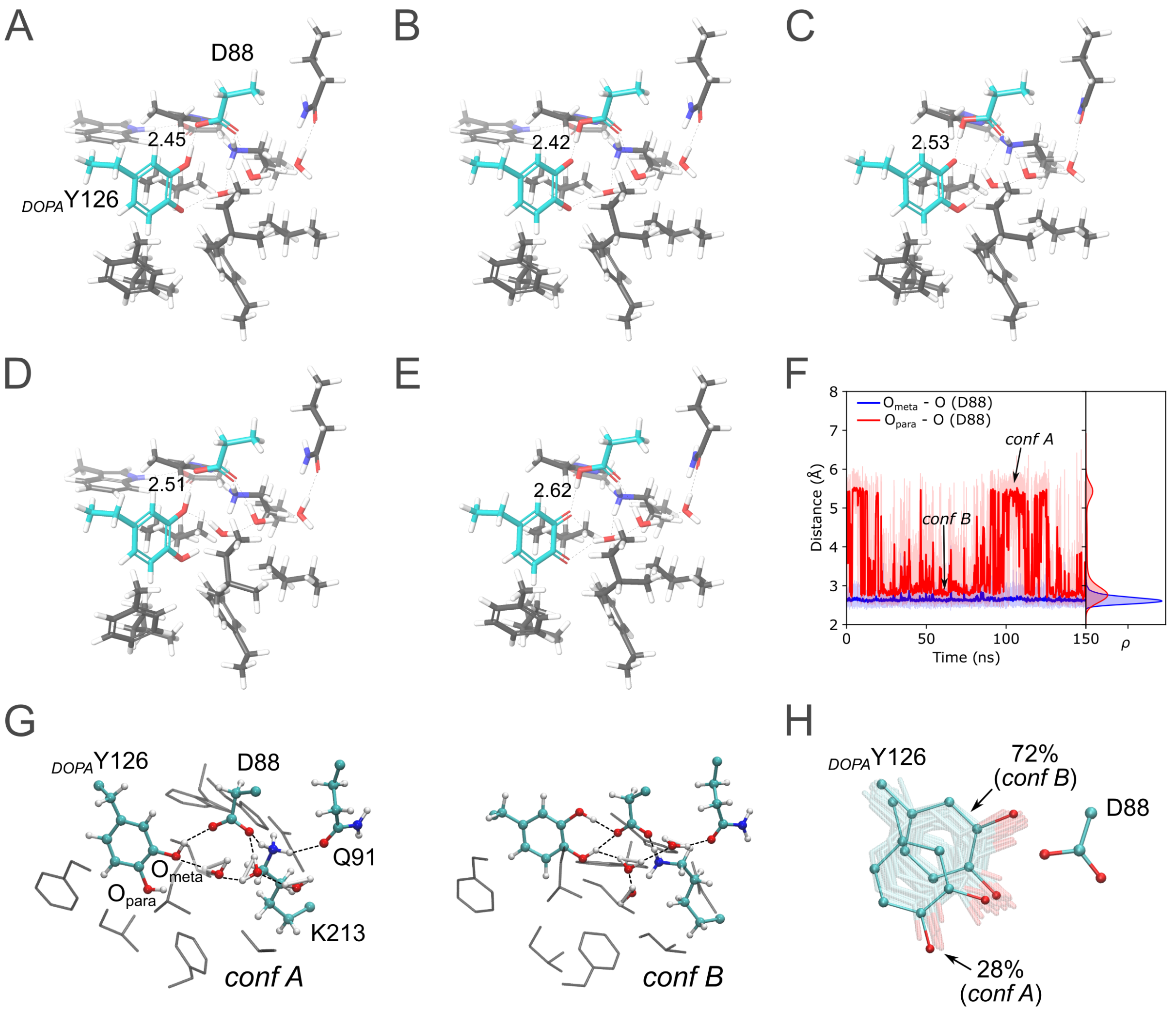

Fig. S3. Theoretical rationalization of the radical and radical-lost states. The short H-bond between $_{DOPA}$Y126 and D88 (residues colored in cyan) suggests a radical state (**A**-**E**). Based on quantum mechanical calculations on the XFEL structure the experimentally observed short hydrogen bond between $_{DOPA}$Y126 and D88 can be reproduced by a DOPA radical state with the radical located on *para*-O (**A**) and (**B**). A proton transfer between $_{DOPA}$Y126 and D88 results in a neutral (**A**) and negatively charged (**B**) DOPA radical state respectively, with similar energy. A DOPA radical state with the radical character on *meta*-O, and protonated D88 shows a longer hydrogen bond (**c**). A neutral DOPA forms a longer hydrogen bond with D88 (**D**). The two-electron oxidized quinone state forms a longer hydrogen bond to D88 than experimentally observed (**E**). Molecular dynamics (MD) simulations starting in the radical state XFEL structure suggest that $_{DOPA}$Y126 with lost radical moves into the position found in the radical-lost crystal structure (F to G). The dominant conformational state of $_{DOPA}$Y126 (*conf B*) forms two hydrogen-bonds with D88, whereas *conf A* only forms one hydrogen-bond with D88. Time evolution of para and meta oxygen atoms of $_{DOPA}$Y126 with D88 during MD **(F)**. Snapshots of the two conformations of $_{DOPA}$Y126 and their interaction with the surrounding protein residues and water molecules **(G)**. Conformational sampling of $_{DOPA}$Y126 during the MD **(H)**. Nitrogen and oxygen atoms are shown in blue and red, respectively. Carbons are shown in grey or cyan. Hydrogen-bonding interactions are indicated by black dashed lines and O–O distances are in Å.

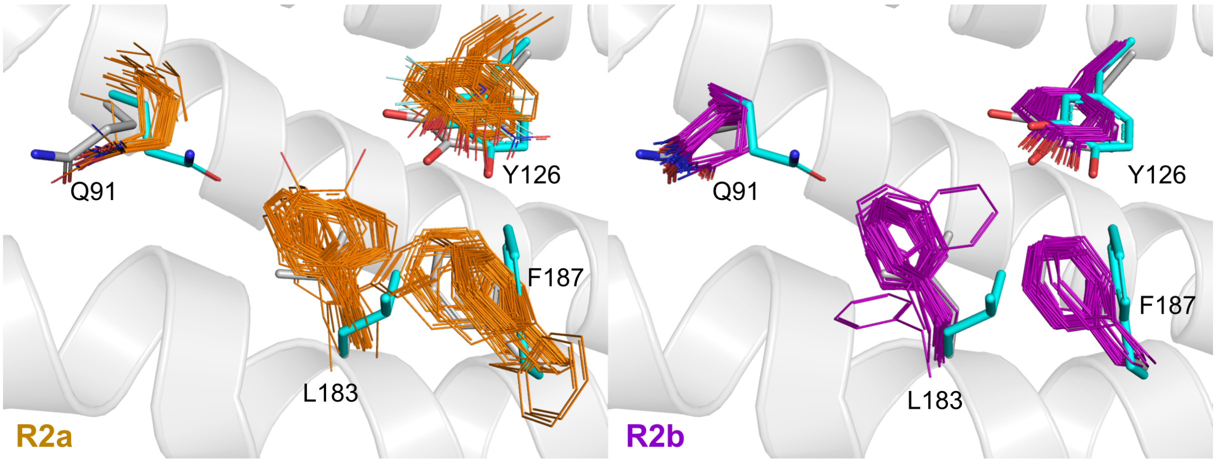

Fig. S4. Comparison of the *Mf*R2e structures to all published R2a and R2b structures in the PDB with resolution of 2.5 Å or better. All structures were aligned with PyMOL to the ground state structure and equivalent residues to Q91, Y126, L183 and F187 of *Mf*R2e are shown in lines; R2a structures are shown in orange on the left side and R2b in purple on the right side. The radical and ground state *Mf*R2e structures are shown in cyan and grey sticks, respectively. For clarity, helices of *Mf*R2e in the ground state only are shown in the background as cartoon. Residues Q91, L183 and F187 exhibit unique conformations in the *Mf*R2e radical structure in comparison with all other structures. PDB IDs of aligned structures are for R2a: 6sf5, 3vpn, 3vpo, 3olj, 1h0n, 1h0o, 1w68, 1w69, 1xsm, 4djn, 1piy, 1piz, 1pj0, 1pj1, 1pm2, 1jpr, 1jqc, 1mxr, 1pfr, 1pim, 1piu, 1r65, 1rib, 1rnr, 1rsr, 1rsv, 2av8, 1yfd, 1biq, 5ci0, 5ci1, 5ci2, 5ci3, 5ci4, 1smq, 2o1z, 1xik, 2xof, 7bet, 7ai8, 7ai9, 6zjk, 7aik, 7ail, 7q39,7q3c; and for R2b: 6tqv, 6tqw, 6tqx, 6tqy, 6tqz, 6qo5, 6qo7, 6qo8, 6qo9, 6qob, 4bmq, 4bmr, 4bmt, 4bmu, 3mjo, 3n37, 3n38, 3dhz, 1kgn, 1kgo, 1kgp, 1oqu, 1r2f, 1uzr, 2r2f, 4dr0, 4n83, 4m1f, 2bq1, 6mw3, 4bmo, 4bmp, 3n39, 3n3a, 3n3b, 7z3d, 7z3e.

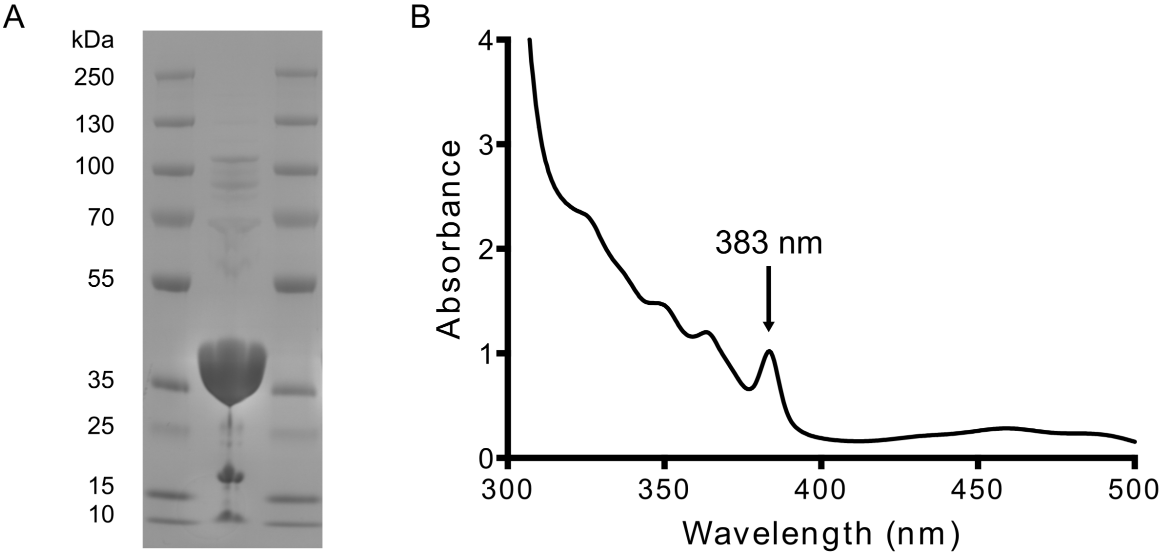

Fig. S5. Purity and radical content of *Mf*R2. (**A**) SDS-PAGE showing the purity of *Mf*R2. (**B**) The UV–visible spectrum of the blue-colored protein shows a peak at 383 nm and additional structure at lower wavelengths characteristics of the catalytically active radical state of *Mf*R2 (*13*).

Table S1. Data collection and refinement statistics

	XFEL radical state *Mf*R2	Synchrotron radical-lost state *Mf*R2
**Data collection**		
Wavelength (Å)	1.30177	1.0
Space group	*C*2	*C*2
Cell dimensions		
a, *b*, *c* (Å)	142.99 ± 0.36<br>46.31 ± 0.10<br>58.51 ± 0.18	175.50<br>53.37<br>79.03
α, β, γ (°)	90, 112.59, 90	90, 108.38, 90
Resolution (Å)	66.01 - 1.50 (1.53 - 1.50)	48.62 - 1.35 (1.398 - 1.35)
Unique reflections	56800 (2710)	144603 (13258)
Completeness (%)	99.97 (100.00)	94.89 (87.47)
Multiplicity	49.2 (19.9)	4.3 (4.3)
$I / \sigma I$	52.3 (1.5)	14.72 (1.88)
R_{split} (%)	23.7 (96.0)	-
R_{meas} (%)	-	5.3 (93.94)
$CC_{1/2}$ (%)	93.1 (34.4)	99.9 (74.3)
Wilson B-factor	20.48	18.49
**Refinement**		
Resolution (Å)	23.82 - 1.50 (1.554 - 1.50)	48.62 - 1.35 (1.398 - 1.35)
No. reflections	56747 (5592)	144555 (13239)
R_{work} / R_{free}	0.1787 (0.3137) / 0.2133 (0.3363)	0.1432 (0.2619) / 0.1640 (0.3015)
No. atoms	2714	6074
Protein	2580	5439
Ligand/ion	0	44
Water	134	591
B-factors	28.84	25.27
Protein	28.37	23.96
Ligand/ion	-	46.43
Water	37.79	35.78
R.m.s. deviations		
Bond lengths (Å)	0.006	0.005
Bond angles (°)	0.78	0.74
Ramachandran (%)		
Favored	99.01	99.34
Outliers	0.00	0.00
Rotamer outliers (%)	0.71	0.51
Clashscore	0.78	1.38

Values in parentheses are for highest-resolution shell.

Captions for Movies S1 to S2

Movie S1: Active site structure with electron density maps for radical and radical-lost states.

Movie S2: Overview and comparison of radical and radical-lost states with local and global structural rearrangements.
